# Adaptive-Neuro-Fuzzy-Based Information Fusion for the Attitude Prediction of TBMs

**DOI:** 10.3390/s21010061

**Published:** 2020-12-24

**Authors:** Boning He, Guoli Zhu, Lei Han, Dailin Zhang

**Affiliations:** State Key Lab of Digital Manufacturing Equipment & Technology, School of Mechanical Science and Engineering, Huazhong University of Science and Technology, Wuhan 430074, China; heboning@hust.edu.cn (B.H.); glzhu@hust.edu.cn (G.Z.); m201970599@hust.edu.cn (L.H.)

**Keywords:** tunnel boring machine (TBM), information fusion, ANFIS, Kalman filter, attitude prediction

## Abstract

In a tunneling boring machine (TBM), to obtain the attitude in real time is very important for a driver. However, the current laser targeting system has a large delay before obtaining the attitude. So, an adaptive-neuro-fuzzy-based information fusion method is proposed to predict the attitude of a laser targeting system in real time. In the proposed method, a dual-rate information fusion is used to fuse the information of a laser targeting system and a two-axis inclinometer, and then obtain roll and pitch angles with a higher rate and provide a smoother attitude prediction. Considering that a measurement error exists, the adaptive neuro-fuzzy inference system (ANFIS) is proposed to model the measurement error, and then the ANFIS-based model is combined with the dual-rate information fusion to achieve high performance. Experimental results show the ANFIS-based information fusion can provide higher real-time performance and accuracy of the attitude prediction. Experimental results also verify that the ANFIS-based information fusion can solve the problem of the laser targeting system losing signals.

## 1. Introduction

**Figure 1:**
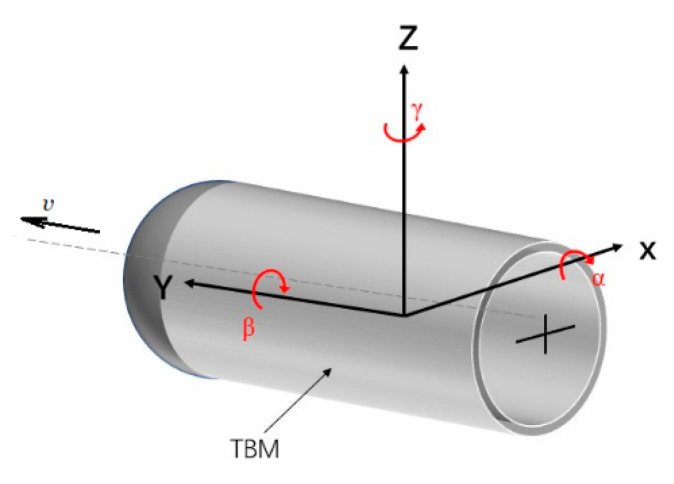
The yaw, pitch, and roll angles of a tunnel boring machine (TBM).

Tunnel boring machines (TBMs) have high work efficiency and safety and are widely used to excavate tunnels, especially for subways, railways, and pipelines [1,2,3,4,5]. During the excavation process, accurate attitude acquisition is very important for controlling TBMs [6,7] and micro tunneling boring machines [8,9]. The attitude consists of three angles, that is, yaw(γ), pitch(α), and roll(β) shown in Figure 1, which are detected by sensors installed at limited observation points on a TBM. In the figure, *v* is the velocity of the TMB. A driver is required to monitor the measured attitude in real time to tune the drive system to ensure the TBM runs in the as-designed tunnel alignment.

The tunnels for subways are more than tens of kilometers long, so the general excavation process is that two TBMs work simultaneously in opposite directions towards one point to improve efficiency. In this situation, the accuracy of the TBM’s guidance system is crucial to steer the TBM to run along the as-designed tunnel alignment and avoid big deviations from the design.

Laser-based targeting systems [9,10,11,12] are widely applied to TBMs as guidance systems. In a laser-based targeting system, a laser theodolite or a laser station needs to be installed in the jacking shaft to detect the position and attitude of the TBM. From the station, the laser beams are projected onto a target board mounted on a TBM, then the attitude angles can be obtained by analyzing the laser spots on the target board. In addition, a two-axis gravity-referenced inclinometer is used to help obtain the pitch and roll angles. The inclinometer can achieve a high sampling rate, which is much higher than that of a laser-based targeting system. However, inclinometers are vulnerable to the strong vibrations resulting from complex geological conditions [13,14,15]. Laser-based targeting systems have high anti-vibration ability but low sampling rate. So, it is feasible to fuse these two measurement methods to predict the attitude accurately with a high sampling rate and anti-vibration ability.

Many methods have been investigated to fuse the information of different sensors [16,17,18,19,20]. D–S evidence theory is a promising and popular approach to data fusion, which can deal with the uncertainty and inconsistency of multi-sensor data, and handle the inevitable ambiguity and instability under noise or possible interference [21]. Similar to the D–S evidence theory, the possibility theory and fuzzy set theory are good methods to deal with imperfect data in the information fusion [22,23].

The Kalman filter [24,25,26,27] is one of the most widely used methods because of its simplicity and effectiveness. A Kalman-based approach [27] could be used to estimate the state and force of a rotating helicopter blade. There are many methods used to improve the performance of the basic Kalman filter. For example, the extended Kalman filter (EKF) [28] can be applied to nonlinear systems while the basic Kalman filter is suitable for linear systems, and the unscented Kalman filter [29] achieved better performance than that of the EKF at a comparative level of complexity. An adaptive cubature Kalman filter [30] was proposed to solve the problem of an initial misalignment angle with uncertain noise covariance matrices for the inertial navigation system. In most cases, the Kalman filter can be used to filter noises during the information fusion, and in some cases, the Kalman filter is used to fuse the information with different sampling rates. For multi-rate cases, dual-rate Kalman filters [31,32] were developed to fuse the sensors with different rates and improve measurement performance.

For some complex situations, adaptive methods [33,34,35] and neural networks [36] can be used in the information fusion to improve the fusion effectiveness. For example, an adaptive federated Kalman filter (FKF) with time-varying information sharing factors [34] was proposed to improve the accuracy, robustness, and fault-tolerance ability of unmanned ground vehicles (UGV). A progressive LiDAR adaptation-aided road detection (PLARD) approach [35] was proposed to adapt LiDAR information into visual image-based road detection. An ensemble convolutional neural network model [36] was proposed to solve the problem of information losses during the information fusion. Hybrid fusion approaches are a comprehensive scheme, which combine different fusion methods such as fuzzy reasoning, D–S evidence theory, and neural networks to complete complex fusion tasks [37,38,39,40]. An adaptive fuzzy extended Kalman filter [41] was developed for attitude estimation with the outputs of strap-down IMU (gyroscopes and accelerometers) and strap-down magnetometer.

This paper proposes an adaptive-neuro-fuzzy-based (ANFIS-based) information fusion system to improve the attitude prediction accuracy of TBMs. Firstly, a dual-rate information fusion is used to reduce the delay of signal sampling and then improve the prediction accuracy of a TBM’s attitude. It is common for one of the fused sensors to have a big error resulting in a bad fusion effect. In our experiments, the roll and pitch angles of a laser targeting system and a two-axis inclinometer were fused, and the two-axis inclinometer had a big measurement error. Secondly, adaptive neuro-fuzzy inference systems are proposed to build the model of the measurement error. Although it can be corrected by tuning the installation attitude, the measurement error cannot be eliminated because of the installation error, the temperature variation, the complex TBM structure, and so on. The measurement error from the two-axis inclinometer will reduce the fusion effect during the information fusion, so the measurement error needs to be predicted and compensated for. The neural networks and fuzzy inference systems are often combined with Kalman filters to optimize the filtering process by building complexing system models [42,43,44]. Adaptive neuro-fuzzy inference systems (ANFIS) [45,46] with the advantages of the neural networks and fuzzy systems can realize both the modeling of the strong nonlinear system and a real-time update of system parameters. Considering the laser targeting system has high accuracy, the pitch angle obtained from the laser targeting system is used as a reference signal to correct the measurement error of the two-axis inclinometer, and an ANFIS is proposed to build the model of the measurement error and update the model according to the real-time data. Finally, the compensated pitch angle is used for information fusion.

The main contribution of the paper is that an adaptive-neuro-fuzzy-based information fusion is proposed to improve the attitude prediction accuracy of TBMs. Compared with the traditional dual-rate information fusion, the ANFIS can be used to build the measurement error model of the pitch angle by the two-axis inclinometer and thus be combined with the traditional dual-rate information fusion to improve the prediction accuracy of the TBM’s pitch angle. Another contribution of the paper is that ANFIS-based information fusion can solve the problem of the laser targeting system losing its signals.

The paper is organized as follows: Section 2 presents the dual-rate information fusion for the attitude prediction; Section 3 describes an ANFIS-based information fusion method. Section 4 and Section 5 give the experimental results and conclusions, respectively.

## 2. Information Fusion for the Attitude Prediction

### 2.1. Attitude Measurement of a TBM

**Figure 2:**
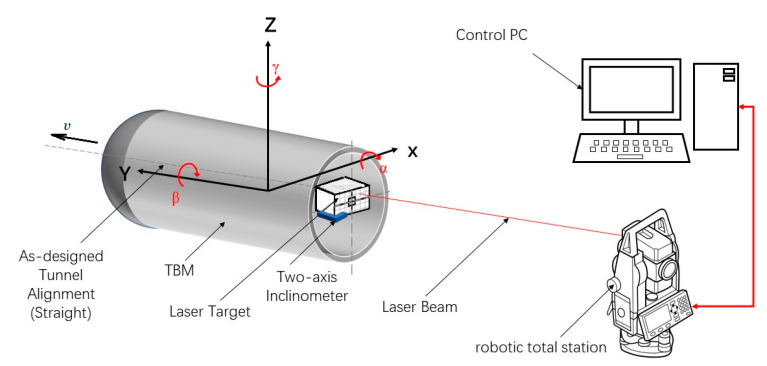
Laser targeting system.

Figure 2 shows the schematic diagram of the used laser targeting system, where α, β, and γ are pitch, roll, and yaw angles, respectively. The TBM runs along the direction of the velocity *v*. A robotic total station projects laser beams onto the laser target, and a camera is used to generate the image of the target board to compute the attitude. When the TBM moves, the robotic total station can track the laser target by tuning its attitude and obtain the distance information according to the feedback laser beam. Computers connect the robotic total station and the two-axis inclinometer to sample the position and attitude information. By analyzing the image from the camera and combining the attitude of the robotic total station, the attitude of the TBM can be obtained (please see Ref. [47] for the details).

Generally, the laser targeting system has high accuracy and anti-vibration ability when it obtains the attitude. However, it is time-consuming to track the laser target, decouple, and correct every attitude angle, so the laser targeting system has a low sampling rate. Sometimes the laser beam is interrupted by some unexpected objects, which will result in the loss of the attitude data. To meet the requirements of high accuracy and a high sampling rate, the information fusion method is proposed in the paper.

The two-axis inclinometer has a very high sampling rate, which is much higher than that of the laser targeting system. However, the two-axis inclinometer is vulnerable to the vibration of the TBM and thus has low accuracy. In this section, the two-axis inclinometer and laser targeting system are fused to take the advantages of the two measurement methods.

### 2.2. Dual-Rate Information Fusion for the Attitude Prediction

**Figure 3:**
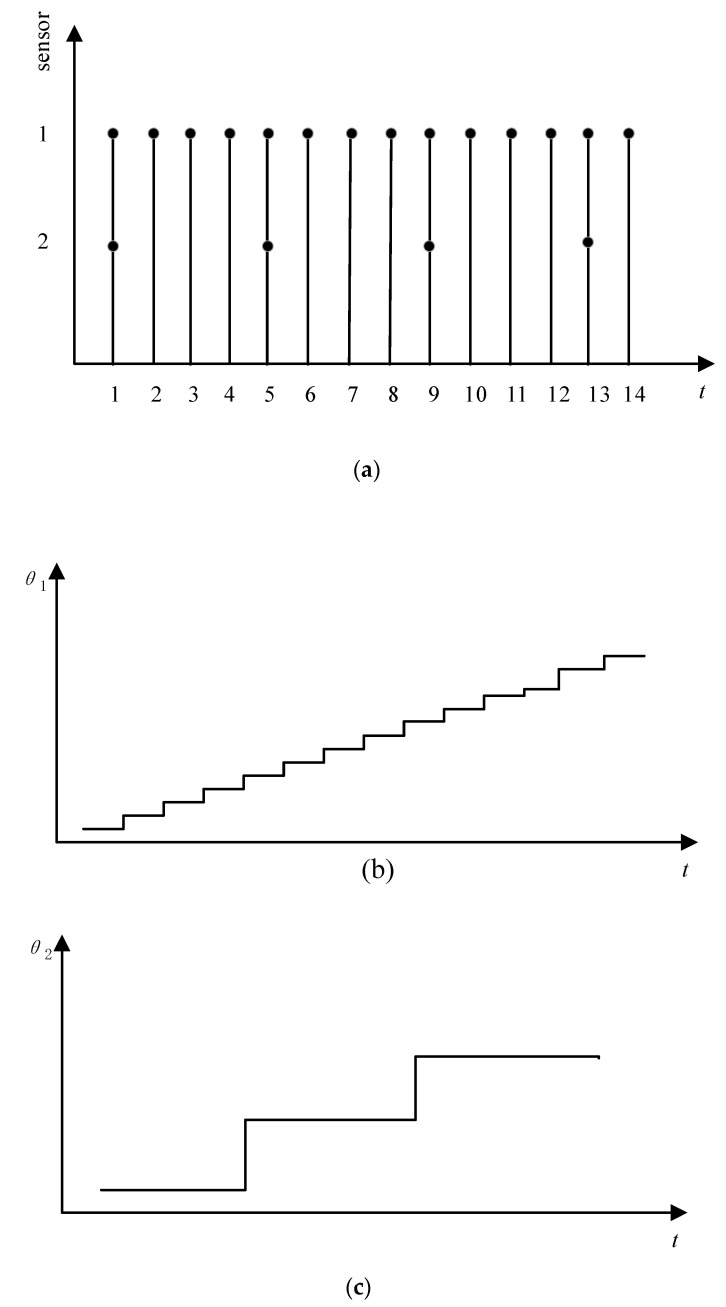
Dual-rate sampling: (**a**) sampling case of sensors; (**b**) attitude angle observed by sensor 1; (**c**) attitude angle observed by sensor 2.

Dual-rate information fusion is often used to fuse information with different rates [32,48]. Figure 3 shows the scheme of the dual-rate sampling. Sensors 1 and 2 in Figure 3a show sampling cases of the attitude angles by the laser targeting system and inclinometer. The attitude angles sensed by the inclinometer (*θ*_1_) and the laser targeting system (*θ*_2_) are shown in Figure 3b,c, respectively. The sampling period of *θ*_1_ is four times that of *θ*_2_. The stairs shown in Figure 3c are longer than those in Figure 3c, which implies that the angle needs to wait for a longer time to be updated, which is not beneficial for a driver to achieve the real-time attitude information. The attitude angle by the inclinometer has a high sampling rate and a low delay, but it is vulnerable to noises. By the following dual-rate information fusion, the two angles can be integrated, and a smooth angle prediction can be obtained to guide the driver to maneuver the TBM.

The state equation of every attitude angle is described as shown in Equations (1) and (2)

(1) X(k+1)=ΦX(k)+ΓW(k)

(2) Y(k+1)=HX(k)+V(k)

where *k* is the discrete time, and X(k)∈R is the state at time *k*. Φ and H are the state transfer matrix and observation matrix, respectively. Γ is the noise matrix. W(k) and V(k) are the input noises and measurement noises, respectively.

The Kalman filter is used to predict the new state by using Equation (3)

(3) $$\hat{X}(k+1|k)=\Phi \hat{X}(k|k)$$

where $\hat{X}(k+1|k)$ is the prediction value of X(k+1|k).

The prediction value of the covariance is obtained by Equation (4)

(4) $$P(k+1|k)=\Phi P(k|k)\Phi^{T}+\Gamma Q\Gamma^{T}$$

where Q is the square error of input noises.

The gain matrix of the Kalman filter is shown in Equation (5)

(5) $$K(k+1)=P(k+1|k)H^{T}{\left[HP(k+1|k)H^{T}+R\right]}^{-1}$$

where R is the square error of measurement noises.

The state update is shown in Equation (6)

(6) $$\hat{X}(k+1|k+1)=\hat{X}(k+1|k)+K(k+1)\varepsilon (k+1)$$

where $\varepsilon (k+1)=Y(k+1)-H\hat{X}(k+1|k)$.

The covariance is updated by Equation (7)

(7) $$P(k+1|k+1)=[I_{n}+K(k+1)H]P(k+1|k)$$

$\alpha_{L}(k)$ and $\beta_{L}(k)$ are the pitch and roll angles sensed by the laser targeting system, and $\alpha_{I}(k)$ and $\beta_{I}(k)$ are the pitch and roll angles, respectively, sensed by the two-axis inclinometer. The four variables can be filtered by the above Kalman filter separately, then the input X(k) in Equation (1) is a one-dimensional vector for every variable. Because the TBM moves slowly, Γ, Φ, and H can be set to 1.

Suppose that the input attitude angles are written as follows:

$$X_{a}(k)=[\alpha_{L}(k)\;\alpha_{I}(k)]$$

$$X_{b}(k)=[\beta_{L}(k)\;\beta_{I}(k)]$$

It is noticed that the $\alpha_{L}(k)$ and $\beta_{L}(k)$ are updated every *n* periods, $\alpha_{I}(k)$ and $\beta_{I}(k)$ are updated every period *T*.

By using Equations (1)–(7), every element of $X_{a}$ and $X_{b}$ can be filtered. It is supposed that the filtered angles are $\alpha_{L\_f}(k)$, $\beta_{L\_f}(k)$$\alpha_{I\_f}(k)$, and $\beta_{I\_f}(k)$, respectively.

With the following fusion matrices, which are Equations (7) and (8), information fusion can be completed.

(8) $$K_{fa}=\left[\begin{array}{l} k_{f1}\\ 1-k_{f1} \end{array}\right]$$

(9) $$K_{fb}=\left[\begin{array}{l} k_{f2}\\ 1-k_{f2} \end{array}\right]$$

where $k_{f1}\in [0,1]$ and $k_{f2}\in [0,1]$ are the fusion gains for pitch and roll angles, respectively.

By using $X_{a}K_{fa}$ and $X_{b}K_{fb}$, the information fusion of the laser targeting system and the two-axis inclinometer can be conducted and the results are Equations (10) and (11)

(10) $$\alpha (k+1|k+1)=k_{f1}\alpha_{L\_f}(k+1|k+1)+(1-k_{f1})\alpha_{I\_f}(k+1|k+1)$$

(11) $$\beta (k+1|k+1)=k_{f2}\beta_{L\_f}(k+1|k+1)+(1-k_{f2})\beta_{I\_f}(k+1|k+1)$$

α(k+1|k+1) can be rewritten as α(k+1). Based on the above information fusion, the one-step prediction values of roll and pitch angles are obtained by the linear prediction Equations (12) and (13)

(12) $$\hat{\alpha }(k+1)=\alpha (k){+k}_{p}(\alpha (k)-\alpha (k-1))$$

(13) $$\hat{\beta }(k+1)=\beta (k){+k}_{p}(\beta (k)-\beta (k-1))$$

where $k_{p}$ is equal to 1. $k_{p}$ can also be any positive proportional coefficient used to predict the future angles at time (*k*+$k_{p}$).

Fusion processes in Equations (10) and (11) require the attitude angles by the laser targeting system and the inclinometer to be approach actual values. In fact, $\alpha_{L}(k)$ and $\beta_{L}(k)$ are much accurate than $\alpha_{I}(k)$ and $\beta_{I}(k)$ at the sampling instants. $\alpha_{I}(k)$ and $\beta_{I}(k)$ are not very accurate with the influence of the vibration, the installation error, the temperature change, and so on. So, the following ANFIS-based information fusion method is proposed to improve the fusion effect.

## 3. ANFIS-Based Information Fusion Method

In TBMs, laser targeting systems have high accuracy and anti-vibration ability and are widely used. So, the attitude obtained by a laser targeting system can be considered to be accurate. The two-axis inclinometer is used to obtain more high-frequency pitch and roll angles. The two-axis inclinometer can act as a master sensor to provide the related angles when the beams of the laser targeting system are not able to be tracked because of sheltering, out of tolerance of roll angle, and so on.

The attitude angles obtained by the two-axis inclinometer need to be corrected. The roll angle has less influence on a TBM running along the as-designed tunnel alignment, so the roll angle obtained by the two-axis inclinometer can be used to act as the master sensor directly after being corrected.

The pitch angle is one of the most important angles to guide a driver to master the TBM. The pitch angle obtained by the two-axis inclinometer is too complicated to be corrected by simply tuning its installation attitude. Its influence factors are so complicated that it is not feasible to model a simple linear model, and the changing influence factors result in model uncertainty. The ANFIS can be used to build a strong nonlinear model and update the model by tuning the system parameters in real time.

### 3.1. ANFIS-Based Information Fusion Method

**Figure 4:**
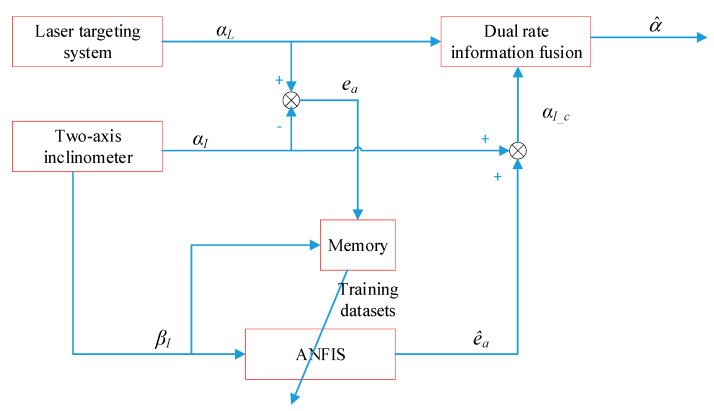
Adaptive neuro-fuzzy inference system (ANFIS)-based information fusion method.

Figure 4 shows the scheme of the ANFIS-based information fusion method. The information fusion method includes two main portions: the dual-rate information fusion and ANFIS. The dual-rate information fusion is used to fuse the pitch angles by the laser targeting system and by the two-axis inclinometer, and the ANFIS is used to model the measurement error to compensate for $\alpha_{I}(k)$.

Because the pitch angle obtained by the laser targeting system is more accurate than that obtained by the two-axis inclinometer, the pitch error $e_{a}$, which is obtained by the difference between $\alpha_{L}$ and $\alpha_{I}$, can be estimated and used to compensate $\alpha_{I}$. According to our experimental analysis, the most important source of the pitch error is the installation error, and the pitch error changes with $\beta_{I}$ and is influenced by the environmental temperature and other factors. So, the idea is to use ANFIS to model the function of $e_{a}$ about $\beta_{L}$ and use the built model to estimate the pitch error in real time. Because $\beta_{L}$ can be replaced by $\beta_{I}$ the built function is suitable to use $\beta_{L}$ to estimate $e_{a}$. By using obtained samples, ANFIS is trained to obtain the optimal estimate ${\hat{e}}_{a}(k)$. The dataset ${\left[e_{a}(k)\;\beta_{I}(k)\right]}^{T}$ is sampled and stored in memory in real time. The new dataset combines the historical datasets to update the parameters of the ANFIS model to adapt to changes of the model.

In the end, the compensated pitch angle $\alpha_{I\_c}$ can be used to fuse with $\alpha_{L}$. The detailed fusion process is shown as follows.

$\alpha_{L}(j)$ and $\alpha_{I}(k)$ are obtained from the laser targeting system and the two-axis inclinometer, respectively. The relation between *k* and *j* is Equation (14)

(14) k=m×j

where *k*, *j* and *m* are positive integers.

Assume the sampling period of the two-axis inclinometer is *T*, then that of the laser targeting system is *mT*. From a driver’s view, the observation value of the laser targeting system will remain within a period of *mT* and the result is shown in Figure 3. Then, $\alpha_{L}(j)$ can extend its values to have the same sampling period as $\alpha_{I}(k)$ by Equation (15)

(15) $$\alpha_{L}(m\times j+i)=\alpha_{L}(j)$$

where i = 1, 2, …, *m*−1.

By the dual-rate information fusion in Section 2, the pitch angle can be obtained by Equation (10). However, $\alpha_{I}(k)$ is not very accurate, so an ANFIS-based information fusion method is proposed as follows.

Taking the pitch angle obtained by the laser targeting system as the reference, the pitch error of $\alpha_{I}(k)$ can be obtained by Equation (16)

(16) $$e_{a}(j)=\alpha_{L}(j)-\alpha_{I}(m\times j)$$

where $e_{a}(j)$ is the pitch error at time *j*.

By using a dataset $E={\left[e_{a}(j)\;\beta_{L}(j)\right]}^{T}$, the model of $e_{a}$ about $\beta_{L}$ is obtained by an ANFIS and $e_{a}(k)$ can be predicted by Equation (17)

(17) $${\hat{e}}_{a}(k)=f(\beta_{I}(k))$$

f(⋅) is the nonlinear function expressed by the ANFIS. By using the prediction ${\hat{e}}_{a}(k)$, $\alpha_{I}(k)$ can be corrected by Equation (18)

(18) $$\alpha_{I\_c}(k)=\alpha_{I}(k)+{\hat{e}}_{a}(k)$$

where $\alpha_{I\_c}(k)$ is the correction of $\alpha_{I}(k)$.

By the dual-rate information fusion in Section 2, with $\alpha_{I\_c}(k+1|k+1)$ replacing $\alpha_{I}(k+1|k+1)$, the fused pitch angle in Equation (10) is revised as Equation (19)

(19) $$\alpha (k+1|k+1)=k_{f1}\alpha_{L\_f}(k+1|k+1)+(1-k_{f1})\alpha_{I\_c\_f}(k+1|k+1)$$

where $\alpha_{I\_c\_f}(k+1|k+1)$ is the filtered $\alpha_{I\_f}(k)$.

The final prediction output of the ANFIS-based information fusion can be obtained by Equation (12).

### 3.2. Angle Correction by ANFIS

**Figure 5:**
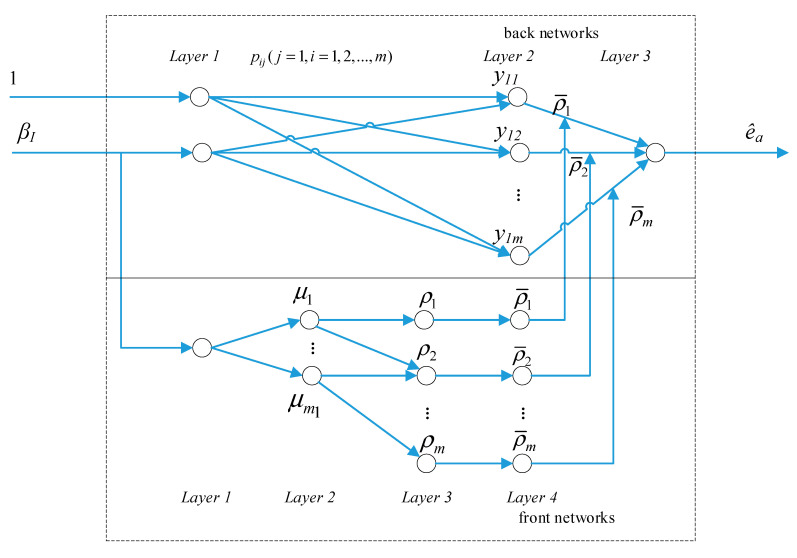
The structure of the single-input single-output Takagi–Sugeno type fuzzy neural networks.

A structure of single-input single-output Takagi–Sugeno type fuzzy neural networks shown in Figure 5 is used in the ANFIS. The whole system is divided into two parts: the front networks and back networks.

The front networks are a Takagi–Sugeno type fuzzy inference system and are composed of four layers as follows.

Layer 1 is the input layer. There is only one input used in the front network of the ANFIS, which is the roll angle $\beta_{I}$.

Layer 2 has the fuzzy variables that are used to compute the membership function of every fuzzy element.

The fuzzy variables of the fuzzy inference system are {NB NM NS O PS PM PB}. For every variable, the membership of every input is $\mu_{i}$, and the Gaussian membership function shown in Equation (20) is applied in the ANFIS

(20) $$\mu_{i}=e^{\frac{\left(x_{i}-c_{i}\right)}{\sigma_{i}}}$$

where $i=1,\;2,\;\ldots ,\;m_{1}$. $m_{1}$ is the number of fuzzy variables. $c_{i}$ and $\sigma_{i}$ are the center and width of the *i*th membership function, respectively.

Layer 3 is the fuzzy rule layer used to compute the fitness of every rule. The following rule shown in Equation (21) is used to compute the fitness of every rule

(21) $$\rho_{i}=\mu_{1}\mu_{2}\cdots \mu_{m_{1}}$$

where i=1, 2, …, m, *m* is the number of the rules, and $\rho_{i}$ is the fitness of every rule.

Layer 4 is the normalization layer used to normalize $\rho_{i}$. The function is shown in Equation (22)

(22) $${\bar{\rho }}_{i}=\frac{\rho_{i}}{\sum_{i=1}^{m}\rho_{i}}$$

where ${\bar{\rho }}_{i}$ is the normalization of $\rho_{i}$.

In the end, ${\bar{\rho }}_{i}$ is used in the back networks to compute the final output.

The back networks are composed of the following three neural network layers.

Layer 1 of the back networks is the input roll angle $\beta_{I}$ and a constant input ‘1′ is used to generate the fixed bias of the *i*th note $p_{10}^{i}$ at Layer 2.

Layer 2 has *m* notes corresponding to *m* rules. The output of the *i*th note is the function of $\beta_{I}$, which is described in Equation (23)

(23) $$y_{1i}=p_{10}^{i}+p_{11}^{i}\beta_{I}$$

where i=1,2,…,m.

Layer 3 is the output of the ANFIS and the output is the estimated pitch error in Equation (24)

(24) $${\hat{e}}_{a}=\sum_{j=1}^{m}\rho_{j}y_{1j}$$

$p_{1m}^{i}$, $c_{i}$, and $\sigma_{i}$ need to be learned by the neural networks.

The cost function is Equation (25)

(25) $$E=\frac{1}{2}{\left(t_{1}-y_{1}\right)}^{2}$$

where $t_{1}$ and $y_{1}$ are the expected and actual outputs of the ANFIS, respectively. In the applied ANFIS, $y_{1}$ is equal to ${\hat{e}}_{a}$.

By using the ANFIS, the model of the measurement error of the pitch angle $e_{a}(k)$ about the roll angle $\beta_{L}(k)$ can be built and updated. By using the generalization ability, $\beta_{L}(k)$ can be substituted by $\beta_{I}(k)$ to obtain a higher rate estimation value ${\hat{e}}_{a}(k)$.

## 4. Experimental Results

**Figure 6:**
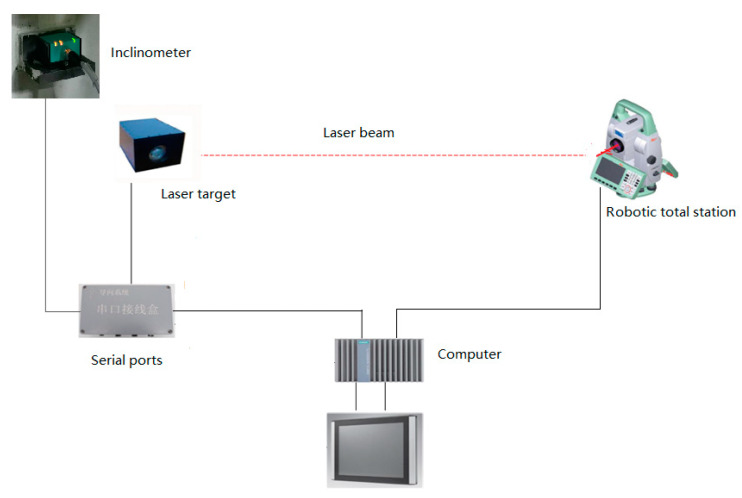
Laser targeting system used in the experiment.

Experiments were conducted to verify the proposed ANFIS-based information fusion. The laser targeting system is shown in Figure 6. The robotic total station projects beams onto the laser target to obtain the attitude information, and the two-axis inclinometer is used to obtain the roll and pitch angles, the attitude information from the two sensors can be transmitted into the computer. The sampling periods of the laser targeting system and the two-axis inclinometer are 2 min and 0.5 min, respectively.

**Figure 7:**
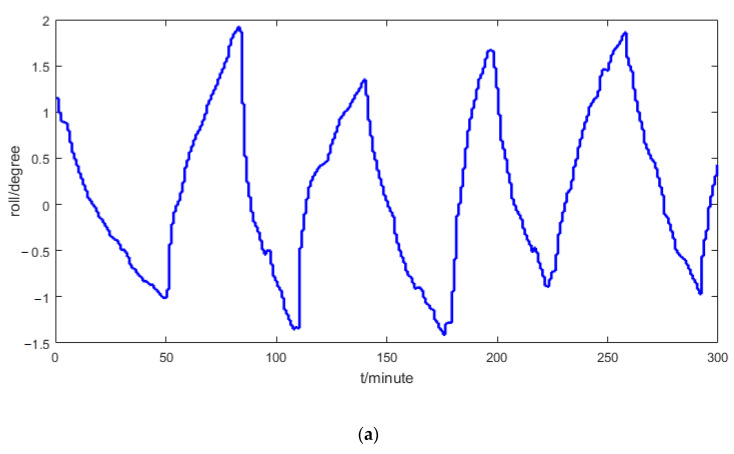

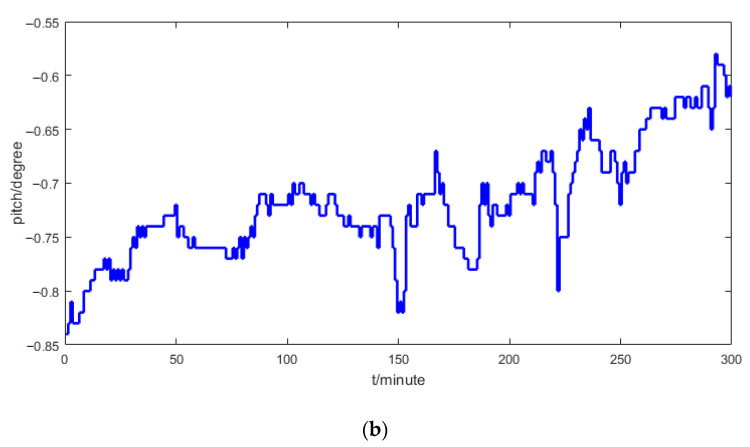
Sampled pitch and roll angles: (**a**) roll, (**b**) pitch.

Figure 7 shows the experimental results of the roll and pitch angles. From a driver’s view, the pitch and roll angles will remain unchanged within sampling periods, which are 2 min and 0.5 min, respectively. From Figure 7, it can be seen that the pitch and roll angles sampled from the laser targeting system have large delays, which will have a big influence on the observation accuracy of the pitch and roll angles.

### 4.1. Roll Angle Prediction

**Figure 8:**
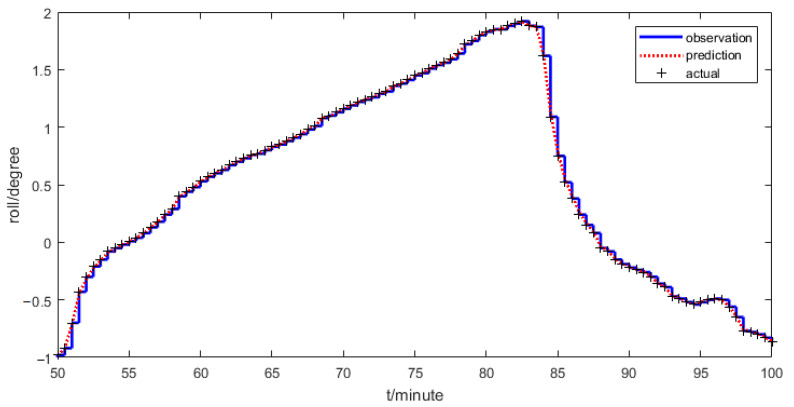
Observed and filtered roll angles.

Figure 8 shows that the laser target system has a low sampling rate to obtain the roll angle labeled as “observation”. The points labeled as “actual” are the values at the sampling instants. Further experiments show that the two-axis inclinometer has a high sampling rate. Because the roll angle does not require very high accuracy and the two-axis inclinometer has a small influence on the roll angle direction, in the dual-rate information fusion $k_{f2}$ is set for 0, which implies that the two-axis inclinometer is directly used to obtain the roll angle at a high sampling rate instead of the dual-rate information fusion. A traditional Kalman filter is used to smooth the roll angle and then the prediction method in Equation (12) is used to obtain the roll angle at any next time. The prediction value of the roll angle is labeled as “prediction”. Compared with the actual value, the observation value has a big error when it is close to the next sampling time, but the prediction value is very close to the actual value.

### 4.2. Pitch Angle Prediction by the ANFIS-Based Information Fusion

**Figure 9:**
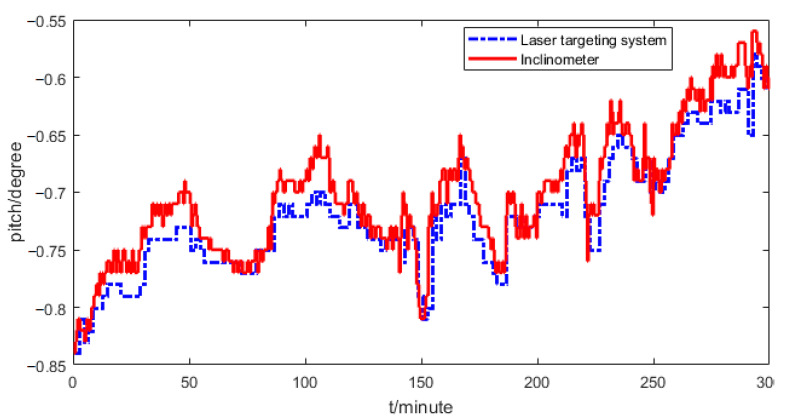
Observed pitch angles.

The dual-rate information fusion method in Section 2 was used to predict the pitch angle. The pitch angles sampled by the laser targeting system and by two-axis inclinometer are shown in Figure 9. The pitch angle obtained by the laser targeting system has a high accuracy with strong anti-vibration ability but a big delay, that obtained by the two-axis inclinometer has a high sampling rate. However, the figure shows that there is a big error between the two pitch angles, so the measurement error needs to be compensated for first before fusing the two sensors.

**Figure 10:**
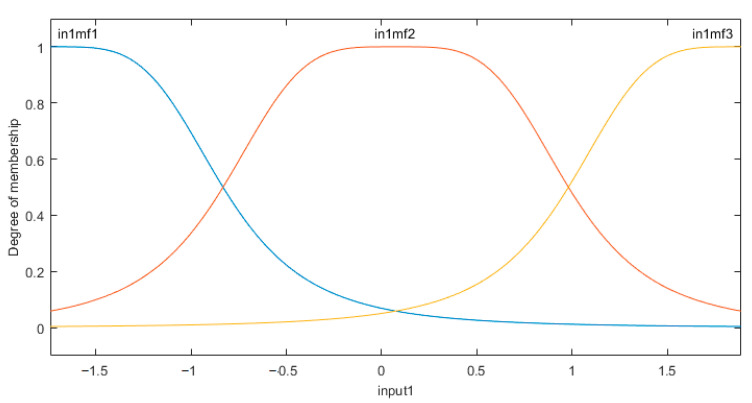
Membership function of the ANFIS.

According to the experimental analysis, the main influence factor of the measurement error is the installation error, and the measurement error is the nonlinear function of the roll angle. The two-axis inclinometer has a simple structure, which is easy to installed, but its correcting process is complex, and the perfect correct result is very difficult to obtain. So, this paper proposes to use an ANFIS to build the nonlinear model of the measurement error. In the system, single-input single-output Takagi–Sugeno type fuzzy neural networks are built and the curves of the membership function in the ANFIS are shown in Figure 10. There are three fuzzy variables used, which are NM, O, and PM. With one input labeled as “in1”, the corresponding memberships are mf1, mf2, and mf3, respectively.

**Figure 11:**
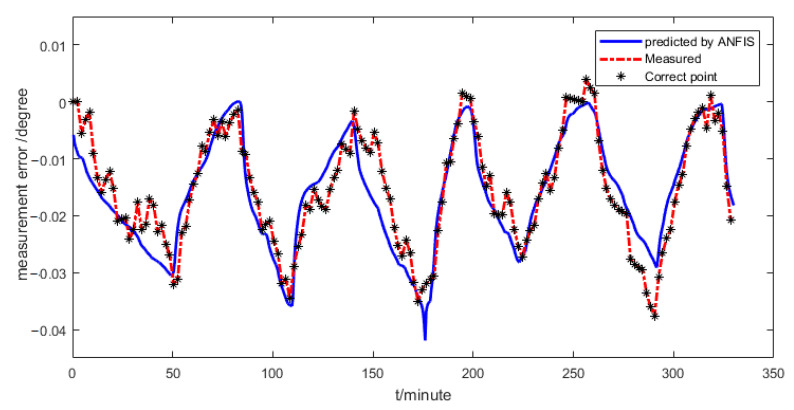
Measurement error of the pitch angle predicted by ANFIS.

From the prediction result shown in Figure 11, it can be seen that the prediction error is smaller than 0.01 degree. The predicted measurement error is used to compensate for the inaccuracy of the two-axis inclinometer, which will improve the fusion accuracy of the two sensors.

**Figure 12:**
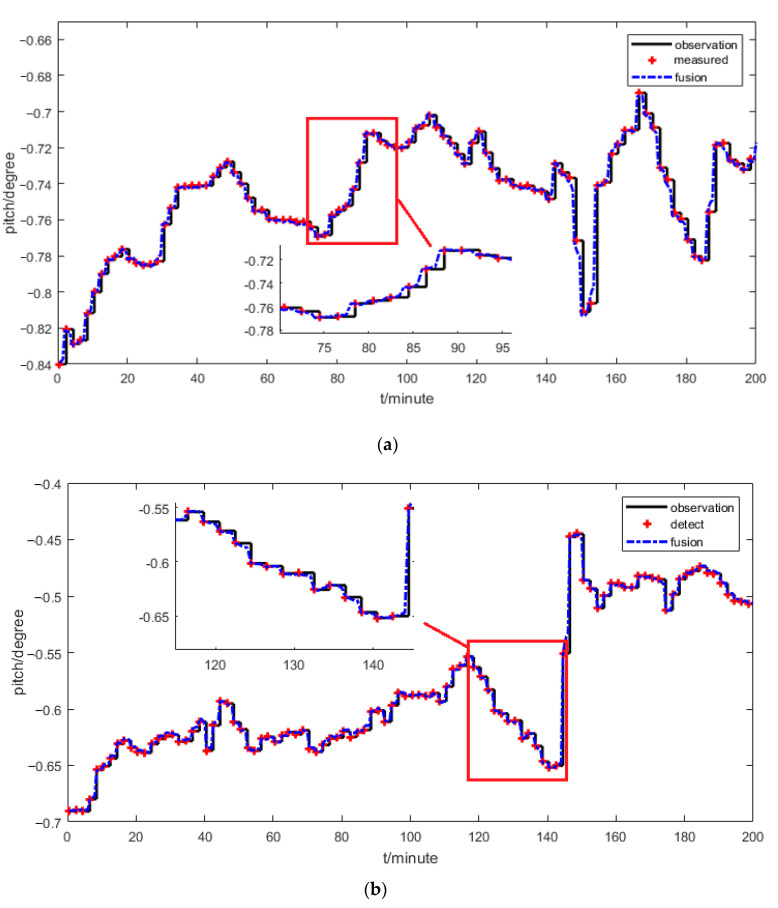
ANFIS-based information fusion for the pitch angle prediction at different times: (**a**) data 1; (**b**) data 2.

Finally, the ANFIS-based information fusion method shown in Figure 4 is used to predict the pitch angle. In this method, the dual-rate information fusion is used to improve the real-time performance and prediction accuracy of the pitch angle, and the fusion gain $k_{f1}$ is set for 0.02. The ANFIS is updated in real time to adapt the complex variation of the measurement error model. Based on the information fusion of the two sensors, the prediction method in Equation (11) is used to predict the pitch angle in real time and the result is shown in Figure 12. Data 1 and data 2 are the data at different times. The sampled points by the laser targeting system labeled as “measured” are used to verify the prediction effect. The results for data 1 and data 2 show that the observation value has a big delay and then a large error near the next sampling point, but the ANFIS-based information fusion can achieve a high sampling rate and a smooth pitch curve. The prediction value at the sampling instant is close to the measured value near the next sampling point, which is smaller than 0.01 degree and much better than the observed value. From the result, it can be concluded that the proposed fusion method can obtain the pitch angle accurately.

### 4.3. ANFIS-Based Information Fusion for Solving the Problem of Signal Losses

**Figure 13:**
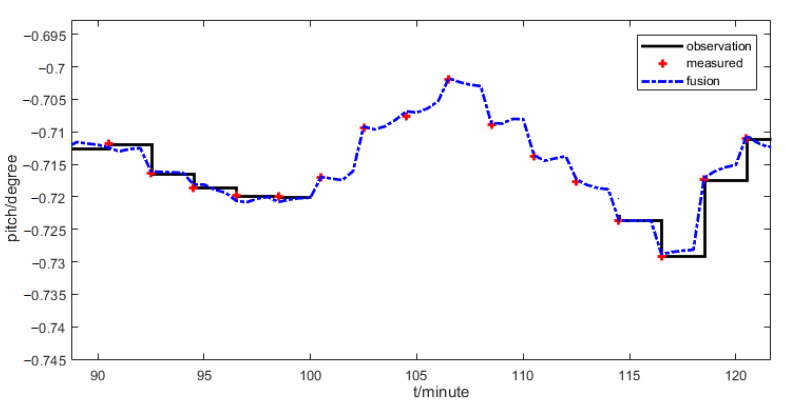
ANFIS-based information fusion when the laser targeting system losses its signals.

Sometimes the laser targeting system loses its signals because of sheltering, being out of tolerance of the roll angle, and so on. The ANFIS-based information fusion can be used to solve this problem. The data in Figure 13 were used to verify its ability to solve the problem.

It is supposed that the data from the laser targeting system are lost from 100 min to 112 min. The ANFIS-based information fusion is used to predict the pitch angle, and when the laser targeting system loses its signals, $k_{f1}$ is set for 0, which implies that the information from the laser targeting system is neglected. In this case, the parameter updating process of the ANFIS stops, then the ANFIS works with fixed parameters.

The result shows that the prediction value by the proposed information fusion has a maximum error of 0.001 degree compared with the measured value. So, the proposed information fusion can solve the problem that the laser targeting system loses its signals.

In summary, the dual-rate information fusion method can realize the information fusion of the laser targeting system and two-axis inclinometer, which can use the advantages of the two sensors and obtain the accurate roll and pitch angles in real time. ANFIS-based information fusion can obtain the accurate pitch angle based on the compensation of the measurement error of the low accurate sensor and can solve the problem of the laser targeting system losing its signals.

## 5. Conclusions

It is very important to obtain the attitude of a TBM in real time. However, the laser targeting system takes a long period to sample the attitude. The inclinometer has a high sampling rate, but it is easily influenced by vibration. To combine the advantages of the two sensors, this paper proposes to use dual-rate information fusion to obtain high real-time and high accurate pitch and roll angles.

The pitch angle has a big measurement error because of the installation error, the temperature variation, the complex TBM structure, and so on, and it is very difficult to be fully corrected: An ANFIS-based information fusion is proposed to predict the pitch angle of TBMs. The ANFIS-based information fusion is mainly composed of the dual-rate information fusion and an ANFIS. The dual-rate information fusion is used to improve the sampling rate and measurement accuracy, and the ANFIS is used to compensate for the measurement error of the low-accuracy sensor.

Experiments were performed to verify the performance of the proposed information fusion method. In the experiments, the pitch angle obtained by the two-axis inclinometer had a big error, so the ANFIS was used to model the pitch error and compensate for the pitch angle. The ANFIS realized the real-time update to adapt the environmental variation. After the pitch angle sampled by the two-axis inclinometer was compensated for, the dual-rate information fusion was used to fuse the two pitch angles.

Experimental results show that the dual-rate information fusion can realize the information fusion of the laser targeting system and two-axis inclinometer, which can use the advantages of the two sensors and obtain the accurate roll and pitch angles in real time. The proposed ANFIS-based information fusion method can obtain a higher accuracy of the pitch angle after compensating for the measurement error. The ANFIS-based information fusion can solve the signal loss problem when the laser beams of the laser targeting system are sheltered or the roll angle is out of tolerance.
